# Psychosocial outcomes and health service use after notifying women participating in population breast screening when they have dense breasts: a BreastScreen Queensland randomised controlled trial

**DOI:** 10.5694/mja2.52117

**Published:** 2023-09-26

**Authors:** Brooke Nickel, Nick Ormiston‐Smith, Lisa Hammerton, Erin Cvejic, Paul Vardon, Zoe Mcinally, Paula Legerton, Karen Baker, Jennifer Isautier, Emma Larsen, Michelle Giles, Meagan E Brennan, Kirsten J McCaffery, Nehmat Houssami

**Affiliations:** ^1^ School of Public Health the University of Sydney Sydney NSW; ^2^ Cancer Screening Unit, Queensland Department of Health Brisbane QLD; ^3^ Sunshine Coast Service, BreastScreen Queensland Nambour QLD; ^4^ Maroondah BreastScreen, Eastern Health Melbourne VIC; ^5^ The University of Notre Dame Australia Sydney NSW; ^6^ The Daffodil Centre, the University of Sydney and Cancer Council NSW Sydney NSW

**Keywords:** Health services research, Breast neoplasms, Women’s health, Public health, Clinical trials as topic

## Abstract

**Background:**

Robust evidence regarding the benefits and harms of notifying Australian women when routine breast screening identifies that they have dense breasts is needed for informing future mammography population screening practice and policy.

**Objectives:**

To assess the psychosocial and health services use effects of notifying women participating in population‐based breast cancer screening that they have dense breasts; to examine whether the mode of communicating this information about its implications (print, online formats) influences these effects.

**Methods and analysis:**

The study population comprises women aged 40 years or older who attend BreastScreen Queensland Sunshine Coast services for mammographic screening and are found to have dense breasts (BI‐RADS density C or D). The randomised controlled trial includes three arms (952 women each): standard BreastScreen care (no notification of breast density; control arm); notification of dense breasts in screening results letter and print health literacy‐sensitive information (intervention arm 1) or a link or QR code to online video‐based health literacy‐sensitive information (intervention arm 2). Baseline demographic data will be obtained from BreastScreen Queensland. Outcomes data will be collected in questionnaires at baseline and eight weeks, twelve months, and 27 months after breast screening. Primary outcomes will be psychological outcomes and health service use; secondary outcomes will be supplemental screening outcomes, cancer worry, perceived breast cancer risk, knowledge about breast density, future mammographic screening intentions, and acceptability of notification about dense breasts.

**Ethics approval:**

Gold Coast Hospital and Health Service Ethics Committee (HREC/2023/QGC/89770); Sunshine Coast Hospital and Health Service Research Governance and Development (SSA/2023/QSC/89770).

**Dissemination of findings:**

Findings will be reported in peer‐reviewed journals and at national and international conferences. They will also be reported to BreastScreen Queensland, BreastScreen Australia, Cancer Australia, and other bodies involved in cancer care and screening, including patient and support organisations.

**Trial registration:**

Australian New Zealand Clinical Trials Registry ACTRN12623000001695p (prospective: 9 January 2023).

Breast density — the ratio of fibro‐glandular tissue to fatty tissue — is an independent risk factor for breast cancer.^1^ Although the influence of some drugs has been investigated,^2^ breast density is less modifiable than other risk factors, such as body mass index and alcohol consumption.^3^ It is estimated that one‐quarter to one‐half of women of breast screening age have heterogeneously or extremely dense breasts;^4^, ^5^ the proportion varies with the age of the women screened (density declines with age^6^) and how breast density is measured and classified.^4^, ^5^ As tumours and fibro‐glandular tissue both appear white in mammograms, high density breast tissue reduces mammographic sensitivity and consequently increases the likelihood that breast cancer is diagnosed between routine screening mammography appointments.^7^, ^8^

Since 2020, United States legislation requires that women and their physicians be notified and appropriately advised about mammographic breast density findings, including their implications for cancer detection.^9^ While notification of breast density is not legally required in other countries, there has been a continuous stream of research into its impact on women, their care providers, and health systems.^10^, ^11^, ^12^ Communication strategies for improving outcomes have been evaluated,^13^ but randomised controlled trial evidence has not been reported. The optimal management of women with dense breasts following notification has been discussed, but consensus regarding the benefits and harms of supplementing mammography with ultrasound or magnetic resonance imaging (MRI) screening is limited.^14^ Although these modalities can detect some cancers not detected in dense breasts by mammography, evidence for long term health benefits or adverse consequences, such as health inequality, has not been reported.^15^ The readability, accessibility, and acceptability of breast density education and information resources have also been scrutinised, often finding that material for women with a lower level of health literacy is needed.^13^, ^16^, ^17^, ^18^

In Australia, women aged 50–74 years without symptoms of breast cancer are invited to participate in free screening mammography every two years; women aged 40–49 or 75 years or older are also eligible for free screening.^19^ However, except in Western Australia and (more recently) South Australia, breast density is not assessed or notified in population‐based breast screening programs. In its position statement on breast density, BreastScreen Australia (the national screening program) notes that it “will continue to work with women, BreastScreen Australia services and researchers to further develop the evidence base and to pilot notification, using emerging reporting tools and initiatives to ensure that valid, reliable and useful information is provided to women to inform future decision‐making.”^20^

## Aims of the study

Our randomised controlled trial (RCT) will assess the effects on psychosocial outcomes and health services use of notifying women participating in population‐based breast cancer screening about mammographic findings of dense breasts. We will also examine whether the mode of communicating this information and advice about its implications (print, online formats) influences these effects. Our study will provide the first RCT‐based evidence regarding the immediate and longer term effects of notifying women about breast density; this evidence could inform population‐based breast screening practice and policy in Australia and overseas.

## Timetable and study sites

- Design phase: 10 August 2022 – 8 January 2023.
- Commence recruitment: 18 September 2023.
- Complete recruitment: 18 March 2024.
- Pilot RCT phase: 18–22 September 2023.
- Commence data collection for main RCT: 18 September 2023.
- Complete data collection: 18 June 2026.

Our RCT will be undertaken in the BreastScreen Queensland clinics in the Sunshine Coast Hospital and Health Service area. Participants will be recruited at eight BreastScreen Queensland Sunshine Coast services (Nambour, Sunshine Coast University Hospital, Caboolture, Caloundra, Gympie, Maroochydore, Noosaville, and the mobile screening van) during routine mammography screening appointments.

## Methods

### Study population and participant recruitment

The study population comprises women aged 40 years or older who attend BreastScreen Queensland Sunshine Coast services for mammographic screening and are found to have dense breasts. All women in this age group who are booked or who present to participating services for screening from 18 September 2023 (with an anticipated recruitment period of six months) will be invited to participate in the study. Those who consent to participation and are classified after screening as having dense breasts (automated density assessment; Volpara Health) — breast imaging reporting and data system (BI‐RADS) density C (heterogeneously dense) or D (very dense)^21^ — will be included in the RCT. Women who do not have dense breasts — BI‐RADS density A (almost entirely fatty) or B (scattered areas of fibro‐glandular density)^21^ — and any women who are recalled because of screen‐detected abnormalities, report symptoms of breast cancer (or clinical concern is noted by the radiographer at screening), have a personal history of breast cancer, require an interpreter (reported at the screening booking or noted by the screening service), cannot consent to breast screening, or do not have an active mobile phone number or email address will not be included in the study.

### Randomised controlled trial component of the study

Our prospective study will comprise an RCT to assess the effects of notifying women screened in the Australian population‐based breast screening program about dense breast findings, and a longitudinal qualitative sub‐study. Eligible women will be allocated in equal numbers to the three study arms using a random number generator without informing them of their allocation:

- arm 1 (control group): standard BreastScreen Australia care (no notification about breast density);
- arm 2 (intervention group 1): notification about dense breasts in the screening results letter and written health literacy‐sensitive information; or
- arm 3 (intervention group 2): notification about dense breasts in the screening results letter and a link or QR code to online video‐format health literacy‐sensitive information (Box 1).

Box 1 **Overview of our investigation of the immediate and longer term effects of breast density notification for women undergoing routine breast screening at eight BreastScreen Queensland Sunshine Coast services**

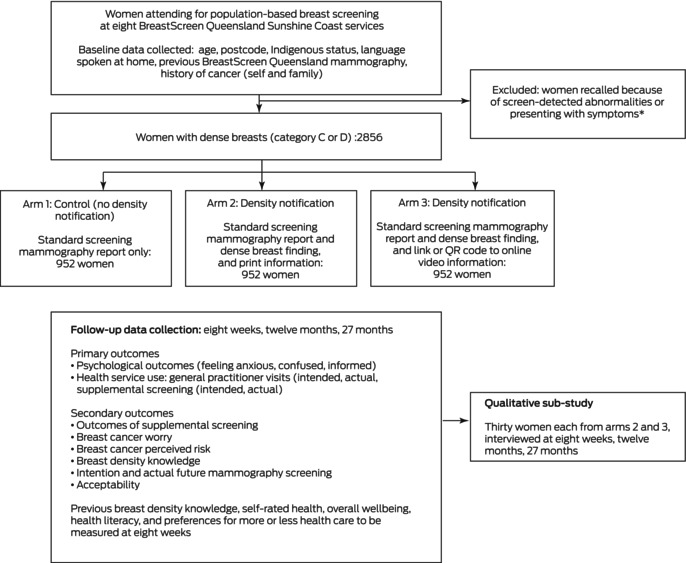

The results of our earlier qualitative studies^22^, ^23^ and experimental RCT^24^ — co‐designed with input from BreastScreen collaborators, as well as local and state community and BreastScreen Queensland panels that included Aboriginal and Torres Strait Islander women — informed the RCT study design and the additional information provided.

The breast density notification letter and additional breast density information will be given to women in arms 2 and 3 of the study with the mammography results report stating that their screening outcome was “no signs of breast cancer detected” (the Well Woman letters sent automatically to all women not recalled because of screen‐detected abnormalities). A short message outlining their breast density result and instructing women to refer to the additional information provided will be included in the Well Woman letters. Women will receive this information according to their pre‐specified information preferences from BreastScreen Queensland by post or via an SMS or email notification to access their results on the BreastScreen Queensland website.

The breast density notification letter will inform women that they have dense breasts and that their doctor can advise them about further tests available to them. Additional detailed health literacy‐sensitive information about what breast density is and its immediate and future implications for the woman will be provided with the notification (Box 2).

Box 2 **Information and advice regarding breast density and its implications provided to women notified that they have dense breasts**

- What are dense breasts?
  ‣ Types of breast tissue
  ‣ Categories of breast density
  ‣ How breast density is measured
- How common is it to have dense breasts?
- Why does breast density matter?
  ‣ Masking
  ‣ Increased risk of breast cancer
- What should I do if a breast screen indicates my breasts are dense?
  ‣ Benefits and harms of supplemental breast cancer screening
  ‣ Be alert to changes in your breasts
  ‣ Talk to your general practitioner

A recent review for BreastScreen Australia recommended that screening information be provided in a variety of formats to allow women to engage with the content as needed and to facilitate decision‐making.^25^ In study arm 2, information about breast density will be included in a separate print document accompanying the notification letter; in arm 3, the notification letter will include an internet link and QR code to a video presenting the same information in a conversational and graphic format.

The nominated general practitioners of the women in study arms 2 and 3 will be forwarded information about the RCT, information about breast density and supplemental screening, and links to the most recent BreastScreen Australia position statement on breast density^20^ and Clinical Oncology Society of Australia breast density information for health professionals.^26^ The study investigators held an information session for Sunshine Coast general practitioners and written in local general practitioner communications about the study, where they provided evidence‐based information on breast density and breast density notification, and answering any questions (further details on trial preparation: Supporting Information).

### Primary and secondary outcomes

Outcomes data will be collected at baseline and eight weeks, twelve months, and 27 months after breast screening (Box 3). This follow‐up period is adequate for estimating subsequent screening behaviour during the recommended two year breast screening interval (27 months according to the BreastScreen Australia national accreditation standard).^27^ Data on socio‐demographic characteristics (age, languages spoken at home, Indigenous status), previous BreastScreen Queensland mammography, history of cancer (self and family), and residential postcode will be obtained at baseline from the BreastScreen Queensland database.

Box 3 **Data collection timetable for our investigation of the immediate and longer term effects of breast density notification for women undergoing routine breast screening at eight BreastScreen Queensland Sunshine Coast services**

**Table:** 

Outcomes	Baseline visit	Immediate (8 weeks)	12‐month follow‐up	27‐month follow‐up
Quantitative outcomes (three research arms, 2856 women)				
Socio‐demographic data, health information	X			
Questionnaire (health descriptors)		X		
Questionnaire (primary, secondary outcomes)		X	X	X
Qualitative sub‐study (arms 2 and 3, sixty women)				
Interview		X	X	X

Primary and secondary outcomes data will be collected from women in all three study arms in identical online questionnaires (Qualtrics) to which they will be directed by an SMS or email link; two reminders will be sent during the week after the first invitation if required. The questionnaire includes validated previously used and study‐specific questions. The primary outcome measures are:

- psychological outcomes (feeling anxious, confused, or informed);^28^ and
- health services use (general practitioner consultations related to breast density; intentions regarding supplemental screening; frequency and modality of supplemental screening).

Secondary measures are the outcomes of supplemental screening (eg, interval cancer diagnosis), worry about breast cancer,^29^ perceived risk of breast cancer,^30^ knowledge regarding breast density,^20^, ^31^, ^32^ intentions regarding frequency of future mammography screening, and acceptability of receiving breast density information with the breast screening results letter.^33^ At eight weeks we will also collect information on prior breast density awareness,^28^, ^34^ self‐rated health,^35^ overall wellbeing,^36^ health literacy,^37^ and preferred frequency of health care^38^ (Box 4). Women in the two intervention arms will also be asked at eight weeks to indicate their willingness to be contacted about the qualitative sub‐study.

Box 4 **Baseline measures, health descriptors, and primary and secondary outcome measures**

- Baseline measures (time of screening)
  ‣ Socio‐demographic characteristics: age, languages spoken at home, Indigenous status.
  ‣ Postcode/Statistical Area Level 2 (SA2).
  ‣ Personal and family history of cancer.
  ‣ Previous mammography with BreastScreen Queensland.
- Health descriptors (8 weeks after screening)
  ‣ Previous knowledge regarding breast density: single item quantitative measure used in other breast density studies.^28^, ^34^
  ‣ Self‐rated health: single item quantitative measure of general health from the 36‐Item Short Form Survey (SF‐36).^35^
  ‣ Overall wellbeing: the 5‐Item World Health Organization Wellbeing Index (WHO‐5) is a validated short questionnaire that measures psychological wellbeing.^36^
  ‣ Health literacy: single item health literacy question, validated using the abbreviated version of the Test of Functional Health Literacy in Adults (S‐TOFHLA).^37^
  ‣ Medical Maximiser–Minimizer Scale (MM1): single item measure of preference for aggressive or passive approaches to health care.^38^
- Primary outcomes (8 weeks, 12 months, 27 months)
  ‣ Psychological outcomes: questions about the psychological impact of knowing one's breast density, adapted from an American survey,^28^ were tested in our earlier experimental randomised controlled trial (RCT).^25^ The psychological outcome was feeling anxious (uneasy, worried, or nervous), informed, or confused.
  ‣ Health service use: a series of questions tested in our earlier experimental RCT^25^ to assess:
  ‣ Intention to consult a general practitioner (8 weeks), number of general practitioner consultations (12 months, 27 months);
  ‣ Supplemental screening intentions (8 weeks), frequency and modality of supplemental screening (12 months, 27 months).
- Secondary outcomes (8 weeks, 12 months, 27 months)
  ‣ Outcomes of supplemental screening (eg, interval cancer diagnosis; 12 months, 27 months).
  ‣ Breast cancer worry: single item used in a British study^29^ and tested in our earlier experimental RCT.^25^
  ‣ Perceived risk of breast cancer: single item.^30^
  ‣ Knowledge regarding breast density: questions adapted from published surveys^28^, ^31^, ^32^ and tested in our experimental RCT.^25^ The questions assess knowledge relevant to decision making, including what breast density means, the increased cancer risk, prevalence, the masking effect, and decline with age.
  ‣ Future intentions regarding mammography screening (8 weeks; 12 months, if applicable) and actual use (12 months, if applicable; 27 months): a series of purpose‐designed questions. Findings of any mammography screening will be collected with these questions and from the BreastScreen Queensland database.
  ‣ Acceptability: summary statistics from the BreastScreen Queensland database for women who declined to participate in (baseline) or who dropped out of the RCT (8 weeks). Questions about acceptability were adapted from a published questionnaire.^33^

### Sample size calculation

We assume that about 15% of women in the control group will report feeling anxious (agree or strongly agree) or use health services (eg, general practitioner consultation or intend to undergo supplemental screening). Achieving 80% power to detect differences in proportions for the primary outcomes between each of the intervention arms and the control arm of ten percentage points (adjusted α = 0.013 for three pairwise comparisons) would require that 373 women in each study arm. We will also undertake longitudinal analyses, and assume 80% retention at eight weeks, 70% at twelve months, and 70% at 27 months. To maintain the overall sample size of 1119 women at 24 months, we aim to recruit 2856 women for the baseline (952 per arm). No further control of the family‐wise error rate was applied in our evidence‐generating real world study.

Based on the number of women screened at the BreastScreen Queensland Sunshine Coast services and a conservative estimate that 30% of screened women will have dense breasts,^39^ recruiting the desired target sample size will take about six months. A brief pilot phase (one week) will ensure that any problems are identified and resolved prior to the main RCT; if no changes to the study design are required, data for the pilot participants will be included in our analysis. If changes are needed, the pilot participant data will not be included, and an application to amend the ethics approval will be submitted.

### Qualitative sub‐study component

Eight weeks after screening, we will purposively recruit (according to a range of socio‐demographic characteristics) sixty women (thirty from each of the two intervention study arms) for interviews by telephone or via Zoom that will be audio‐recorded and transcribed. The interviews will explore personal views regarding the RCT outcome measures over time, including feelings of anxiousness or confusion, interest in and reasons for seeking or not seeking supplemental screening, and the acceptability of breast density notification. The sample size is adequate for a qualitative study.^40^

The qualitative sub‐study will take a phenomenological perspective and the outcomes assessed using thematic analysis.^41^ This method enables themes to be compared both within individuals (eg, a woman's psychological response and understanding of breast density information, and her prior experience and future intentions regarding health service use) and between individuals (eg, women who are or are not anxious).

### Statistical analysis

We will summarise participant socio‐demographic and health characteristics as descriptive statistics (frequencies and proportions for categorical variables; means with standard deviations for continuous variables). Outcomes data by study arm will be analysed in linear mixed models and generalised linear mixed models; the regression models will take correlations between longitudinally collected data (when appropriate) and clustering by recruitment site into account, and allow inclusion of data from individuals for whom some longitudinal outcomes data are missing.

## Ethics approval

The Gold Coast Hospital and Health Service Ethics Committee (HREC/2023/QGC/8977) and Sunshine Coast Hospital and Health Service Research Governance and Development (SSA/2023/QSC/89770) have approved the study.

### Participant consent

Women will be informed in detail about the study prior to and on the day of their appointment, and will be provided a participant information statement and a consent form. The participant information statement will inform them about the aim and design of the study, and advise them that they may receive additional information about their breasts. The participant information statement does not mention the breast density criterion for study participation in order to ensure that participants in the control group are blinded to their breast density status. It will also include contact details for the research team and other support services, and state that participation is voluntary and without penalty should they not participate. Women will be informed that they will be contacted by SMS (or email if they do not have a mobile phone) with links to brief online questionnaires. They will also be informed that some personal information will be obtained from BreastScreen Queensland for study purposes only.

### Data safety

All data will be stored in password‐protected files on password‐protected computers, backed up to a University of Sydney server. Quantitative and qualitative study data will be retained for fifteen and five years, respectively, in accordance with National Health and Medical Research Council guidelines,^42^ after which the files will be deleted.

## Dissemination of findings

Evaluating the effects of notifying or not notifying women about dense breast information using two different modes of communicating will facilitate translation of our findings to BreastScreen Queensland and other screening services. They will be disseminated in publications in peer‐reviewed national and international journals, and presented at national and international conferences. Our findings will also be reported to BreastScreen Queensland, BreastScreen Australia, Cancer Australia, and other national and international bodies involved in cancer care and screening, including those currently considering routine notification of women about breast density and patient and support organisations.

No information that could identify study participants will be included in any presentation of our findings. All quantitative analyses will be of de‐identified data, and audio‐recordings and transcripts will be de‐identified for the qualitative sub‐study. Quantitative findings will be reported at the group level, and individual qualitative findings and quotes will be anonymised.

## Data sharing statement

In line with our ethics approval application, quantitative data will be anonymised and aggregated for statistical analyses, and findings will be reported at the group level. Qualitative findings will also be reported at the group level. Any additional unpublished aggregate‐level data will be available upon reasonable request at the conclusion of the RCT. Individual‐level data and RCT data files will not be accessible to external researchers.

## Trial registration

The RCT was prospectively registered with the Australian New Zealand Clinical Trials Registry on 9 January 2023 (ACTRN12623000001695p).

## Funding statement

Our study is supported by a National Health and Medical Research Council (NHMRC) Emerging Leader Research Fellowship (1194108) for Brooke Nickel, and co‐supported by a National Breast Cancer Foundation Chair in Breast Cancer Prevention grant (EC‐21‐001) to Nehmat Houssami. Kirsten McCaffery (2016719) and Nehmat Houssami (1194410) are supported by NHMRC Investigator (Leader) Fellowships. The funders had and will have no role in the planning, analysis, or publication of the study.

## Author contributions

Brooke Nickel and Nehmat Houssami conceived the study; all listed authors contributed to study design and development; Brooke Nickel drafted the protocol manuscript; and all authors reviewed and approved the final protocol manuscript.

## Open access

Open access publishing facilitated by the University of Sydney, as part of the Wiley – the University of Sydney agreement via the Council of Australian University Librarians.

## Competing interests

No relevant disclosures.

## Supporting information


Supplementary methods
