# Supplementary material for: Psychosocial outcomes and health service use after notifying women participating in population breast screening when they have dense breasts: a BreastScreen Queensland randomised controlled trial
Source: Med J Aust. 2023 Sep 26;219(9):423–8. doi: 10.5694/mja2.52117 (PMC10952548; doi:10.5694/mja2.52117)
Supplement: Supplementary file 1 — Supplementary methods [file MJA2-219-423-s001.pdf]

The Medical Journal of Australia

Supporting Information

Supplementary methods

**This appendix was part of the submitted manuscript and has been peer reviewed.
It is posted as supplied by the authors.**

Appendix to: Nickel B, Ormiston-Smith N, Hammerton L, et al. Psychosocial outcomes and health service use after notifying women participating in population breast screening that they have dense breasts: a BreastScreen Queensland randomised controlled trial. *Med J Aust* 2023; doi: 10.5694/mja2.52117.

Further information on activities related to the preparation of the randomised controlled trial component of the study

- Pre-randomised controlled trial (RCT) consultations with Department of Health & BreastScreen Australia to develop earlier scientific work[1-3] that informed current RCT plans, included presentations to Department of Health (25/03/2021, 27/01/2022) & BreastScreen Australia managers meeting (08/03/2022)
- Co-design of RCT: met 31/05/2022 and 21/06/2022, then commenced weekly or fortnightly meetings from 11/08/2022 until RCT initiation
- Presented to Sunshine Coast Consumer Reference Group (25/11/2022) for feedback on study
- Developed study documents (including intervention letters, questionnaires, participant information statement, consent forms, etc.) in consultation with BreastScreen Queensland, BreastScreen Queensland Sunshine Coast Service
- RCT registered with Australia New Zealand Clinical Trials Registry 9/01/2023
- BreastScreen Queensland Consumer reference group and two local consumers (from Sunshine Coast Consumer Reference Group) viewed and commented on study and related documents
- Ethics submitted to the GCHHS 11/01/2023, approved 6/03/2023
- Volpara density technology installed and tested, and letter process confirmed with BreastScreen Queensland mail house
- The University of Sydney Risk Assessment – approved and rated (moderate) 23/03/2023
- Developed video with BreastScreen Queensland video production team
- Developed Frequently Asked Questions for the service and evidence-based information sheet for general practitioners
- Presented to Local Medical Association (general practitioners) on the Sunshine Coast 25/05/2023, newsletter article written and published ahead of the event
- Presentation distributed among the Sunshine Coast Local Media Association membership and General Practitioner Liaison group in June, July and August 2023 newsletters
- Clinical Trial Risk Assessment and Budget finalised
- Site-Specific Ethics Amendment approved by the SCHHS 3/08/2023
- Training of service staff and radiographers 30/08/2023
- Local Sunshine Coast media coverage 12/09/2023-20/09/2023
- BreastScreen Queensland 'Well Woman' letters updated 13/09/2023

References

1. Nickel B, Dolan H, Carter S, et al. General practitioners' (GPs) understanding and views on breast density in Australia: a qualitative interview study. *BMJ Open* 2021; 11: e047513.
2. Nickel B, Dolan H, Carter S, et al. "It's about our bodies… we have the right to know this stuff": a qualitative focus group study on Australian women's perspectives on breast density. *Patient Educ Couns* 2021; 105: 632-640.
3. Dolan H, McCaffery K, Houssami N, et al. Australian women's intentions and psychological outcomes related to breast density notification and information: a randomized clinical trial. *JAMA Netw Open* 2022; 5: e2216784.
